# Does psychosocial stress exacerbate avoidant responses to cancer information in those who are afraid of cancer? A population-based survey among older adults in England

**DOI:** 10.1080/08870446.2017.1314475

**Published:** 2017-04-09

**Authors:** Charlotte Vrinten, David Boniface, Siu Hing Lo, Lindsay C. Kobayashi, Christian von Wagner, Jo Waller

**Affiliations:** ^a^ Research Department of Behavioural Science and Health, University College London, London, UK; ^b^ Harvard Center for Population and Development Studies, Harvard University, Cambridge, MA, USA

**Keywords:** cancer, fear, worry, psychosocial stress, health communication, avoidance

## Abstract

***Objective:*** Communication of cancer information is an important element of cancer control, but cancer fear may lead to information avoidance, especially when coping is low. We examined the association between cancer fear and cancer information avoidance, and tested whether this was exacerbated by psychosocial stress.

***Design:*** Cross-sectional survey of 1258 population-based adults (58–70 years) in England.

***Main outcome measures:*** Cancer fear (intensity and frequency), perceived psychosocial stress and cancer information avoidance. Control variables were age, gender, ethnicity, marital status and education.

***Results:*** A quarter (24%) of respondents avoided cancer information. Ordinal logistic regression analyses showed main effects of psychosocial stress (OR = 1.17, 95% CI 1.07–1.29) and cancer fear: cancer information avoidance was lowest in those with no cancer fear (13%), followed by those with moderate (24%; OR = 2.15, 95% CI: 1.49–3.12), and high cancer fear (35%; OR = 3.90, 95% CI: 2.65–5.73). In the adjusted model, the interaction between cancer fear and stress was significant (OR = 1.14, 95% CI 1.004–1.29, *p* < .05): 40% of those with high fear/high stress avoided cancer information compared with 29% with high fear/low stress.

***Conclusion:*** Cancer fear and psychosocial stress interact to produce disengagement with cancer-related information, highlighting the importance of affective processes to cancer control efforts.

## Background

Cancer control strategies include public communication on prevention and screening, but in order to be effective these communications need to reach their intended audience (Viswanath, 2005). Individuals may vary in their receptiveness to cancer information, with some likely to be ‘information-seekers’ and some ‘information-avoiders’ (Case, Andrews, Johnson, & Allard, 2005; Miller, 1995; Sweeny, Melnyk, Miller, & Shepperd, 2010). Studies have shown that those who seek cancer information tend to have better cancer knowledge, lower levels of smoking, healthier diet and exercise habits, and higher levels of cancer screening uptake (Kelly et al., 2010; Shim, Kelly, & Hornik, 2006), highlighting the importance of accessing available information. Greater knowledge about cancer, particularly of cancer warning signs, is in turn associated with seeking help for potential cancer symptoms more quickly (De Nooijer, Lechner, & De Vries, 2001; Quaife et al., 2014; Simon, Waller, Robb, & Wardle, 2010). Conversely, cancer information avoidance has been shown to undermine preventive health behaviours, such as cancer screening (Emanuel et al., 2015).

Cancer fear is one factor that could promote cancer information avoidance. According to Witte’s extended parallel process model (EPPM), the effect of fear (operationalised as a negative emotion elicited by threat and accompanied by high arousal) depends on the individual’s access to resources to control the threat (Witte, 1992). When control strategies to mitigate the threat are available, fear motivates appropriate action responses to control the threat (danger control), which, if successful, will allay the fear elicited by the threat. When danger control strategies are not available, fear motivates actions to control the fear itself (fear control); for example, by denial, avoidance or downgrading the importance of the threat (Witte, 1992, 1998). However, if this response does not adequately control the threat, it will continue to elicit fear. Thus, coping strategies play a central role in the EPPM in determining the behavioural response to the fear elicited by a threat. This conceptualisation of resources determining the response to threatening information has also been put forward by other authors using different theoretical underpinnings such as crisis decision theory (for example, Howell, Crosier, & Shepperd, 2014; Sweeny, 2008).

Surveys in the UK and US indicate that cancer fear is prevalent in the general population. Community-based studies show that only about a third of the adult population are *not at all* worried about their risk of getting cancer, about half are *a bit* worried and a minority are *quite* or *very* worried (Wardle et al., 2000). Similarly, studies of cancer worry frequency in the general population show that about half of people *never* or *rarely* worry about their risk of cancer, about a third *sometimes* worry about cancer and a small minority (8%) worry *often* or *all the time* (Han, Moser, & Klein, 2007; Rakowski et al., 2006). Fear of cancer is associated with non-attendance at cancer screening (Clemow et al., 2000; Howell, Shepperd, & Logan, 2013; McLachlan, Clements, & Austoker, 2012), doctor avoidance (Persoskie, Ferrer, & Klein, 2014) and delayed help-seeking for possible cancer symptoms (De Nooijer et al., 2001; Lund-Nielsen, Midtgaard, Rørth, Gottrup, & Adamsen, 2011); all of which can be framed as cancer avoidance behaviour. In addition to behavioural avoidance, cancer fear also promotes cognitive avoidance: higher levels of cancer fear are associated with higher levels of cancer information avoidance (Miles, Voorwinden, Chapman, & Wardle, 2008; Nelissen, Beullens, Lemal, & Van den Bulck, 2015), and poorer recall of cancer-related information (Miles, Voorwinden, Mathews, & Wardle, 2007).

Psychosocial stress can also lead to avoidance. Psychosocial stress has been defined as ‘a process in which environmental demands tax or exceed the adaptive capacity of an organism, resulting in psychological or biological changes that may place a person at greater risk for disease’ (Cohen, Kessler, & Gordon, 1995). Stress has been shown to reduce health information seeking (Case et al., 2005), and to promote general health care avoidance (Ye, Shim, & Rust, 2012). Stress may promote avoidance by causing cognitive and emotional depletion, thus reducing coping resources and motivating the individual to focus on the more immediate stressors and avoid situations that might add further stress (Case et al., 2005; Gallo & Matthews, 2003). Thus, psychosocial stress may shift responses towards the fear control processes of the EPPM, rather than the danger control processes. No studies have examined a potential relationship between stress- and cancer-related information avoidance. However, previous research has shown reasons such as being ‘too busy’, ‘not having got round to it’ or having ‘too many other things to worry about’ are frequently cited for putting off going to the doctor with potential cancer symptoms (Robb et al., 2009; Waller et al., 2009), or attending cancer screening (Lo, Waller, Wardle, & von Wagner, 2013; Waller, Bartoszek, Marlow, & Wardle, 2009).

To date, little evidence exists of how emotional factors may interact to affect behaviour. In this study, we examined associations of cancer fear and stress with cancer information avoidance in a population-based sample of middle-aged and older adults. We hypothesised that both cancer fear and stress would be associated with cancer information avoidance and, based on Witte’s EPPM, we tested the hypothesis that there is an interaction of cancer fear and stress, with the association between fear and avoidance stronger for individuals with higher levels of psychosocial stress. In terms of their sociodemographic distribution, we know that cancer fear is more prevalent in women and those with lower levels of education (Vrinten, van Jaarsveld, Waller, von Wagner, & Wardle, 2014), while stress is more prevalent among men, and those who are younger or from lower socioeconomic status (SES) (Hatch & Dohrenwend, 2007). These emotional factors may thus cluster in certain population subgroups, such as those from lower SES backgrounds, which could make them hard to reach for cancer control strategies that depend on public communication of cancer information.

## Methods

### Sample and data collection

Data come from the Attitudes, Behaviour, and Cancer-UK Survey (ABACUS), a large population-based cross-sectional omnibus survey in England carried out by TNS Research International between January and March 2014. This weekly survey creates sample points using the 2001 Census small-area statistics and the Postcode Address File (stratified by social grade and Government Office Region) for random location sampling. Quotas for age, gender, children in the home and working status are set for each location, and three doors are left between each successful interview. Data were collected using computer-assisted face-to-face interviews by a trained interviewer in the respondents’ homes, as part of a module about attitudes towards colorectal cancer screening using faecal occult blood testing, which was administered to respondents between 58 and 70 years old. NHS ethical approval for this study was obtained (13/NW/0707), and participants consented to participate at the start of the omnibus survey.

### Measures

Cancer information avoidance was measured with three items adapted from a questionnaire developed by the UK Colorectal Cancer Screening Pilot Evaluation Team (UK CRC Screening Pilot Evaluation Team, 2003): ‘*Do you avoid reading stories about cancer in a newspaper, a magazine, or online’*, ‘*Do you avoid watching programmes about cancer on TV*’ and ‘*Do you avoid talking to other people about cancer’*. Response options were ‘yes’ (1), ‘no’ (0), ‘don’t know’ and ‘refused to answer’, in addition to ‘not applicable’ for the two media-related items. The latter three responses were coded as missing. The scale showed good levels of internal reliability (Cronbach’s *α* = .76). For those with complete data on all three items, a sum score was created to indicate levels of avoidance (0–3).

Perceived psychosocial stress was measured using a single item, adapted from Littman, White, Satia, Bowen, and Kristal (2006): ‘*How would you rate the amount of stress in your life* – *this includes at home and/or at work’.* Responses were scored on a six-point Likert scale from 1 ‘no stress’ to 6 ‘extreme stress’.

Cancer fear was assessed using two items developed for this survey. One assessed the *intensity* of cancer fear: *‘How anxious do you feel when you think about cancer’* and was scored on a four-point Likert scale: ‘not at all’, ‘slightly’, ‘quite a bit’ or ‘extremely’. The other item assessed the *frequency* of cancer fear, and was adapted from the Health Information National Trends Survey (HINTS) 3 (National Cancer Institute, 2008): *‘How often do you worry about your chance of developing cancer*’. This item was scored on a five-point Likert scale: ‘never’, ‘occasionally’, ‘sometimes’, ‘often’ or ‘very often’. Spearman’s correlation coefficient between the two items was .57 (*p* < .001). For those with complete data, the items were conceptually categorised to create three groups: ‘no’, ‘moderate’ and ‘high’ cancer fear as shown in Table 1.

**Table 1.**
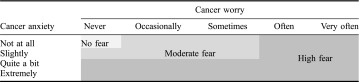
Categorisation of the cancer fear items.

Demographic data included age, gender, educational attainment and marital status. We also assessed ethnicity, but because the majority of our sample (96%) were of white ethnicity (in line with the ethnic distribution of the UK population for this age group; Office for National Statistics, 2005), ethnic differences could not be explored further. Educational attainment was assessed with the question ‘*what is the highest level of educational qualification you have achieved*’. There were eight response options but for analysis they were dichotomised into ‘some educational qualifications’ vs. ‘no educational qualifications’. Educational attainment is a good measure of SES in older adults (Grundy & Holt, 2001). Marital status was assessed using three response options: ‘married or living as married’, ‘single’ and ‘widowed, divorced, or separated’. The latter two were combined into a single category ‘not married or living as married’. For all items, ‘refused’ and ‘don’t know’ responses were coded as missing.

### Statistical analysis

For these analyses, those with a personal history of cancer, as well as those with missing data on cancer information avoidance, cancer fear or perceived stress, were excluded. For the demographic variables, missing cases were coded as a separate category. Descriptive statistics consisted of numbers and percentages, or means and standard deviations, as appropriate. We examined the sociodemographic distribution of perceived stress (using univariate linear regression) and cancer fear (using unadjusted ordinal regression analyses where the assumption of proportional odds was met, or chi square analyses where this assumption was not met). We then examined the effects of cancer fear, perceived stress and their interaction on cancer information avoidance, using ordinal logistic regression analyses and controlling for demographic variables. Ordinal regression has the advantage of maintaining the ordinality of the outcome variable while estimating a single odds ratio to summarise the association of the outcome with the independent variable. In addition, because there is no consensus on how to combine cancer worry and anxiety measures, we conducted three additional analyses: for cancer worry and cancer anxiety separately, and for a sum score of the two items. These results are presented in the online supplement. An alpha level of .05 was used to indicate statistical significance. SPSS version 22.0 was used for all analyses.

## Results

The interview was completed by 1568 respondents, of whom 127 (8.1%) were excluded from the present analyses because of a previous cancer diagnosis. Those with missing data on any of the cancer information avoidance items (*n* = 150; 9.6%), cancer fear (*n* = 27; 1.7%) or perceived stress (*n* = 6; .4%) were also excluded, leaving a final sample of 1258 (80.2% of respondents). Characteristics of the analysed sample are presented in Table 2. The mean age was 64 years (SD = 3.7), and about half were male (51%). About two-thirds had some educational qualifications (66%) and a similar proportion were married or living as married (66%). There were no differences in age, cancer fear or perceived stress between the included and excluded respondents. However, excluded respondents were more likely to be male (59%), and less likely to report any educational qualifications (55%) or to be married or living as married (59%; all *p* < .05).

**Table 2.** Characteristics of the sample (*N* = 1258).

Characteristic	*N* (%)^a^
Age (M, SD)	64.3 (3.7)
Gender	
Male	639 (50.8)
Female	619 (49.2)
Ethnicity	
White	1206 (95.9)
Black and minority ethnic	48 (3.8)
Missing	4 (.3)
Education	
Some qualifications	831 (66.1)
No qualifications	378 (30.0)
Missing	49 (3.9)
Marital status	
Married or living as married	831 (66.1)
Not married or living as married	427 (33.9)
Perceived stress (M, SD)	2.62 (1.35)
Cancer fear	
None	337 (26.8)
Moderate	570 (45.3)
High	351 (27.9)
Cancer information avoidance (yes)	
Reading stories about cancer	202 (16.1)
Watching TV programmes about cancer	241 (19.2)
Talking to others about cancer	104 (8.3)

^a^ Unless otherwise stated.

### Cancer information avoidance

Overall, almost a quarter of the sample (24%) avoided one or more sources of cancer information. One in six respondents (16%) said they avoided reading stories about cancer in a newspaper, magazine or online; 19% avoided watching programmes about cancer on TV; and 8% avoided talking to other people about cancer (Table 2). Across all three avoidance items, about three quarters of the sample (76%) did not avoid cancer information, with a minority avoiding one (10%), two (9%) or all three (5%) sources of cancer information. The results of the unadjusted ordinal logistic regression analyses are presented in Table 3. For ease of interpretation, this table displays the total percentage of those avoiding cancer information, i.e. those avoiding one, two or all three sources of cancer information combined. In terms of sociodemographic distribution, cancer information avoidance was associated with not having any educational qualifications (32% vs. 20% of those with qualifications, OR = 1.92; 95% CI: 1.47–2.52), but not with gender, age or marital status (Table 3).

**Table 3.** Predictors of cancer information avoidance (*N* = 1258).

	Avoids one or more sources of cancer information	Unadjusted ordinal regression
		
	*N* (%)	OR (95% CI)
Age		1.01 (.97–1.04)
Gender		
Male	156 (24.4)	REF
Female	147 (23.7)	.97 (.75–1.26)
Education		
Any qualifications	168 (20.2)	REF
No qualifications	121 (32.0)	**1.92 (1.47–2.52)**
Missing	14 (28.6)	1.72 (.91–3.25)
Marital status		
Married or living as	189 (22.7)	REF
Not married or living as	114 (26.7)	1.30 (1.00–1.70)
Perceived stress		**1.17 (1.07–1.29)**
Cancer fear		
None	43 (12.8)	REF
Moderate	136 (23.9)	**2.15 (1.49–3.12)**
High	124 (35.3)	**3.90 (2.65–5.73)**

Abbreviations: OR = odds ratio; 95% CI = 95% confidence interval. Bold values significant at *p* < .05.

### Perceived psychosocial stress

On average, psychosocial stress was in the lower half of the scale, with a mean score of 2.6/6.0 (SD = 1.35). Univariate regression analyses showed that stress was associated with being younger (*β* = −.144, *p* < .001), being female (M = 2.83, SD = 1.38 for women vs. M = 2.42, SD = 1.29 for men, *β* = .151, *p* < .001), not having any educational qualifications (M = 2.76, SD = 1.50 for those without vs. M = 2.56, SD = 1.27 for those with qualifications, *β* = .068, *p* < .05) and being unmarried (M = 2.87, SD = 1.50 for those who were unmarried vs. M = 2.49, SD = 1.24 for those who were married, *β* = .132, *p* < .001).

### Cancer fear

Most respondents were ‘not at all’ (35%) or ‘slightly’ (39%) anxious when thinking about cancer, 19% were ‘quite’ anxious and 7% were ‘extremely’ anxious. Responses to the frequency item showed that 39% ‘never’ worried about their chance of developing cancer, 32% worried ‘occasionally’, 22% ‘sometimes’, 6% ‘often’ and 1% ‘very often’. For the combined cancer fear measure, about a quarter of the sample (27%) were classified as having ‘no cancer fear’, almost half the sample (45%) had moderate fear and 28% had high cancer fear (Table 2). Unadjusted ordinal regression analyses showed that high cancer fear was positively associated with being female (33% of women vs. 23% of men; OR = 1.59; 95% CI: 1.29–1.95), but not with age or marital status. There were also differences by education: of those without qualifications, 29% had no cancer fear, 37% moderate and 34% had high fear, vs. 26, 49 and 25% of those with educational qualifications, respectively (*χ* ^2^(2)=19.1, *p* < .001).^1^

### Cancer fear and perceived psychosocial stress as predictors of cancer information avoidance

In unadjusted ordinal logistic regression analyses, cancer information avoidance significantly increased with level of cancer fear: total percentages of avoidance across all three items showed that 13% of those with no cancer fear avoided one or more types of cancer-related information, vs. 24% of those with moderate cancer fear (OR = 2.15; 95% CI: 1.49–3.12), and 35% of those with high cancer fear (OR = 3.90; 95% CI: 2.65–5.73). There was also a significant association between psychosocial stress and cancer information avoidance (OR = 1.17; 95% CI: 1.07–1.29).

**Figure 1.**
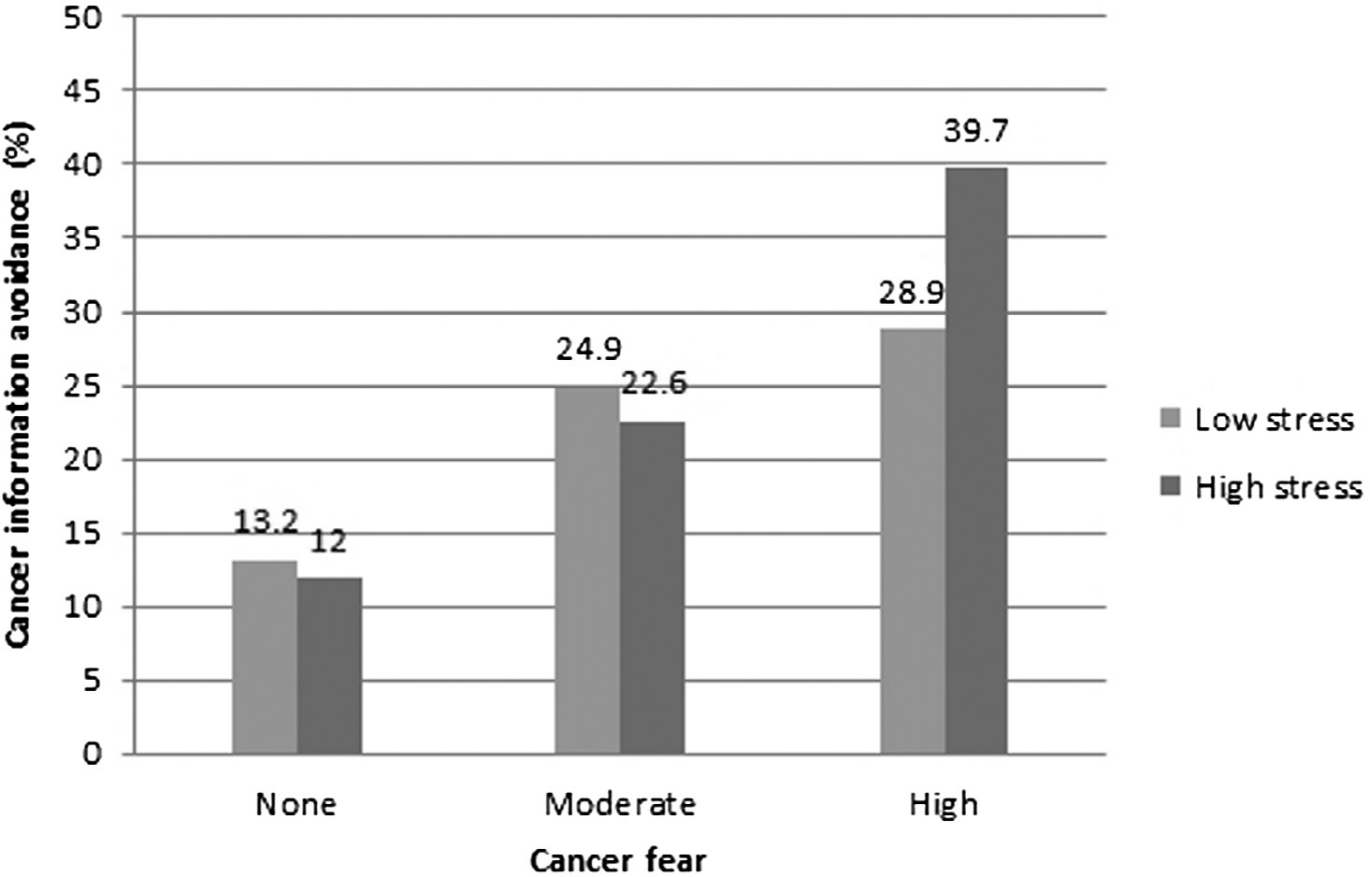
Percentage of respondents avoiding one or more sources of cancer information by level of cancer fear and perceived stress (*N* = 1258).

We then tested the effect of the interaction between cancer fear and perceived stress on cancer information avoidance in a model adjusted for all sociodemographic variables. This interaction term was significant: OR = 1.14, 95% CI 1.004–1.29 (Wald *χ* ^2^(1) = 4.06, *p* < .05). The relationship between cancer fear, perceived stress and cancer information avoidance is illustrated in Figure 1. For ease of interpretation, this figure displays the percentages of respondents avoiding one or more sources of cancer information by level of cancer fear. For purposes of illustration, we also dichotomised perceived psychosocial stress into high or low stress according to the mean. The figure shows higher cancer information avoidance among those with higher cancer fear, with a stronger association for the high stress compared with the low stress group.

## Discussion

Almost a quarter of middle-aged and older adults in the UK avoid some sources of cancer information and, as predicted, those with higher levels of psychosocial stress or cancer fear are more likely to avoid cancer information. The interaction between fear and stress was also significant, with highest levels of avoidance among those with high levels of cancer fear *and* psychosocial stress.

The prevalence of cancer information avoidance in this sample (24%) was similar to a previous community-based study of older adults in the UK (27%; Miles et al., 2008), but may be lower than in the US: population-representative data from HINTS show that 39% of US adults would avoid cancer risk information (Emanuel et al., 2015), and around a third would avoid doctor visits even when they suspect they are needed (Kannan & Veazie, 2014; Persoskie et al., 2014). Our study replicates findings showing that cancer information avoidance is more prevalent among those with higher cancer fear (Miles et al., 2008; Nelissen et al., 2015). Together, these findings provide further evidence that cancer fear is not only related to behavioural avoidance, such as non-attendance at cancer screening (Andersen, Smith, Meischke, Bowen, & Urban, 2003; Vrinten, Waller, von Wagner, & Wardle, 2015; Wong et al., 2013), but also to cognitive forms of avoidance. In addition, our study shows that cancer fear’s association with avoidance is accentuated by high levels of stress, adding to the growing body of evidence of the importance of stress and coping on health, and showing that emotional factors may interact to produce health-related behaviour.

The prevalence of cancer fear in our study was similar to other studies, with about a third not being fearful of cancer, about a third to half slightly fearful and about a quarter to a third quite fearful (Han et al., 2007; Vrinten et al., 2014; Wardle et al., 2000). We also found the same demographic distribution, with women and those with no educational qualifications being more fearful (Vrinten et al., 2014). Perceived psychosocial stress in our sample was slightly lower than in older adults in the general population in the US (Littman et al., 2006), but its demographic distribution in this sample was largely the same, with higher levels of stress in those who were younger, and those from lower SES backgrounds (as indexed by education; Hatch & Dohrenwend, 2007; Littman et al., 2006), and women (Littman et al., 2006).

Stress and fear may thus cluster in women and those from lower SES backgrounds, and this may have implications for the communication of cancer control messages. Although women are less likely to participate in bowel cancer screening (Power, Miles, von Wagner, Robb, & Wardle, 2009), there are no gender differences in cancer information avoidance and help-seeking for cancer symptoms (Macleod, Mitchell, Burgess, Macdonald, & Ramirez, 2009), and they tend to score higher than men on cancer awareness (Robb et al., 2009). However, SES differences in cancer awareness, screening uptake, help-seeking and engagement with cancer information have been well documented (Emanuel et al., 2015; Macleod et al., 2009; Miles et al., 2008; Persoskie et al., 2014; Power et al., 2009; Robb et al., 2009). Avoiding information may be rational in some contexts (Sweeny et al., 2010), but in the context of cancer it may prove detrimental to personal health if it leads to unawareness of cancer warning signs and delays in help-seeking, or non-attendance at cancer screening (Quaife et al., 2014). Disengagement with cancer information may be amplified by cancer fear, which is associated with a negative bias towards interpretation of cancer information (Miles, Voorwinden, Mathews, Hoppitt, & Wardle, 2009), poorer recall of cancer information (Miles et al., 2007) and lower rates of successful cancer information-seeking (Beckjord, Finney Rutten, Arora, Moser, & Hesse, 2008). Our study suggests that fear and stress may cluster in low SES subgroups to produce disengagement with cancer-related information, and may thus help inform efforts to reduce inequalities in cancer outcomes (von Wagner, Good, Whitaker, & Wardle, 2011), including socio-economic inequalities, for this age group who are at high risk of developing cancer.

Our study has several limitations. Due to its cross-sectional design, we cannot draw any conclusions about the temporal stability of stress, fear and avoidance, or the causal relationship between cancer fear and cancer information avoidance. For example, cancer fear may promote avoidance of cancer information, but cancer information avoidance may also perpetuate cancer fear if it prevents people from learning about improved treatment and survival rates for cancer. Longitudinal studies are needed to explore the temporal stability and direction of causality. Furthermore, the fieldwork agency did not provide data on response rates or characteristics of non-responders, and people with higher levels of stress could be under-represented in our sample, if they have chosen not to participate in the interviews. In addition, we had about 10 % missing data on the avoidance items. The percentage of cancer information avoidance may be an underestimate if those who avoid cancer information are also more likely to avoid answering questions about avoidance. Although our cancer information avoidance measure showed good internal reliability, people’s reasons for avoidance were not assessed and the measure may not have picked up on legitimate reasons for failing to engage with cancer information via certain media. A more in-depth investigation of these reasons may be needed.

## Conclusion

Despite cancer information being highly relevant in this age group, a quarter of our respondents said they avoided it. Avoidance was as high as 40% among those who were highly fearful of cancer and perceived their lives to be stressful, showing how affective processes may interact to produce disengagement with health-information. Those from lower socio-economic backgrounds were more likely to feel stressed, be fearful and avoid information about cancer. These findings may have implications for cancer control strategies that rely upon communication of cancer-related information: a significant proportion of the population may avoid this kind of information and any negative views about cancer may remain unchallenged as cancer treatments and prognosis continue to improve. Allaying undue cancer fear and addressing cancer information avoidance could increase engagement with cancer early detection and may help close the socio-economic gap in cancer outcomes.

**Abbreviations**
EPPM: Extended Parallel Process Model
HINTS: Health Information National Trends Survey
NHS: National Health Service
OR: Odds Ratio
SES: Socio-Economic Status

## Authors’ contributions

CV, SHL, LCK, CvW and JW conceived of the study and participated in its design. CV, SHL and DB planned the statistical analyses, which were conducted by CV and DB. CV drafted the manuscript and SHL, LCK, DB, JW and CvW commented on these drafts. All authors read and approved the final manuscript.

## Disclosure statement

No potential conflict of interest was reported by the authors.

## Funding

This work was supported by a grant from Cancer Research UK awarded to Professor Jane Wardle [grant number C1418/A14134].

## Supplemental data

Supplemental data for this article can be accessed here: http://dx.doi.org/10.1080/08870446.2017.1314475

## Supplementary Material

GPSH_1314475_Supplementary_Material.doc

## Note

1. We performed a *χ* ^2^ test rather than an ordinal regression analysis for this variable due to violation of the assumption of proportional odds for this analysis.
