# Supplementary material for: Does psychosocial stress exacerbate avoidant responses to cancer information in those who are afraid of cancer? A population-based survey among older adults in England
Source: Psychol Health. 2017 Apr 9;33(1):117–29. doi: 10.1080/08870446.2017.1314475 (PMC5750809; doi:10.1080/08870446.2017.1314475)
Supplement: GPSH_1314475_Supplementary_Material.doc [file GPSH_A_1314475_SM5928.doc]

**Online supplement to “Does psychosocial stress exacerbate avoidant responses to cancer information in those who are afraid of cancer? A population-based survey among older adults in the UK”** By C. Vrinten, D. Boniface, S.H. Lo, L.C. Kobayashi, C. von Wagner, and J. Waller

**Background**

In the main manuscript, we use a conceptual categorisation to combine the two indicators of cancer fear (i.e. *intensity of cancer anxiety* and *frequency of cancer worry*) into three categories of fear (*no*, *moderate*, and *high cancer fear*) without prior reference to the data to allow for examination of dose-response relationships between cancer fear and cancer information avoidance. However, there is currently no consensus how these two cancer fear indicators can be best combined, and some authors have drawn attention to the disadvantages of categorising continuous variables (e.g. 1, 2). In this online supplement, we therefore present three alternative analyses of the same dataset: for the (A) cancer worry frequency and (B) cancer anxiety intensity items separately, and (C) for a sum score of these items as a continuous variable.

**Methods**

For these analyses, we have used perceived stress, cancer worry, cancer anxiety, and the combination of these two items (‘cancer fear’) as continuous variables. The sum score for cancer fear was created by adding the scores for cancer worry frequency (range: 1-5) and cancer anxiety intensity (range: 1-4). We also created a sum score for cancer fear by adding the standardised z-scores for these items, but these were highly correlated to the sum score created by adding the (non-standardised) raw scores (Pearson’s *r*=.9998, p<.001), so we have chosen to report the analyses using the non-standardised sum score for ease of interpretation (range: 2-9). We then repeated the ordinal logistic regression analyses as reported in the main manuscript.

**Results**

Table. Ordinal logistic regression analyses for perceived stress, cancer worry, cancer anxiety, cancer fear, and the interaction between cancer fear and stress, as predictors of cancer information avoidance (N=1,258). Values in bold are significant at p<.05.

|  | **Unadjusted** | **Adjusted*** |
| --- | --- | --- |
|  | **OR (95% CI)** | **OR (95% CI)** |
| **Model A** |  |  |
| Perceived stress | **1.17 (1.07-1.29)** | 1.00 (0.81-1.23) |
| Cancer worry (1-5) | **1.44 (1.27-1.64)^$^** | 1.21 (0.91-1.62) |
| Cancer worry*stress | **1.09 (1.06-1.11)** | 1.05 (0.97-1.14) |
| **Model B** |  |  |
| Perceived stress | **1.17 (1.07-1.29)** | 0.94 (0.75-1.18) |
| Cancer anxiety (1-4) | **1.70 (1.49-1.96) ^$^** | **1.36 (1.01-1.83)** |
| Cancer anxiety*stress | **1.11 (1.08-1.14)** | 1.08 (0.98-1.18) |
| **Model C** |  |  |
| Perceived stress | **1.17 (1.07-1.29)** | 0.89 (0.69-1.14) **^$^** |
| Cancer fear (sum score; 2-9) | **1.34 (1.24-1.45) ^$^** | 1.16 (0.97-1.38) **^$^** |
| Cancer fear*stress | **1.06 (1.04-1.07)** | 1.05 (0.99-1.11) **^$^** |

*Adjusted for age (continuous), gender, educational level, and marital status.

^$^Violates the assumption of proportional odds for ordinal regression.

These results show that, in all three unadjusted models, cancer worry, cancer anxiety, and cancer fear are positively associated with a higher likelihood of avoiding cancer information, although all three measures of fear violate the assumption of proportional odds for ordinal regression. In addition, their interaction with stress is significant in all three models. This relationship becomes borderline significant in the models mutually adjusted for all variables and demographic differences.

**Conclusions**

Regardless of how cancer fear is measured, there is a positive association of the interaction between cancer fear and perceived psychosocial stress with cancer information avoidance.

**References**

1. Royston P, Altman DG, Sauerbrei W. Dichotomizing continuous predictors in multiple regression: a bad idea. Statistics in medicine. 2006;25(1):127-41.

2. Altman DG, Royston P. The cost of dichotomising continuous variables. BMJ (Clinical research ed). 2006;332(7549):1080.
